# Supplementary material for: Identification of Freezing-Responsive microRNAs and Their Targets in Chinese Jujube by Small RNA and Degradome Sequencing
Source: Int J Mol Sci. 2026 Sep 4;27(17):7912. doi: 10.3390/ijms27177912 (PMC13566890; doi:10.3390/ijms27177912)
Supplement: Supplementary file 1 [file ijms-27-07912-s001.zip › Supplementary Material S2/novel_miR_183.pdf]

Provisional ID	:	novel_miR_183
Score total	:	4.9
Score for star read(s)	:	-1.3
Score for read counts	:	0
Score for mfe	:	4.6
Score for randfold	:	1.6
Score for cons. seed	:	
Total read count	:	50
Mature read count	:	50
Loop read count	:	0
Star read count	:	0

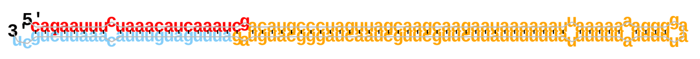

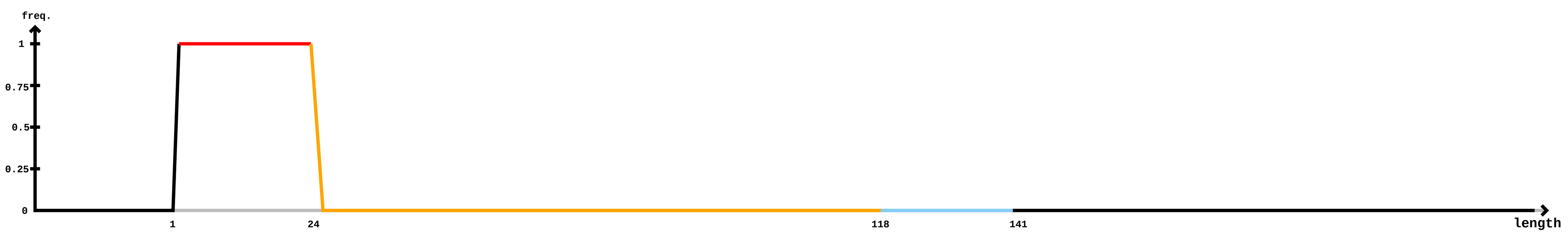


```
                      Mature                                                                                       Star
5'- aaaugugucuggaauagauuagcagaauuucuaaacaucaaaucgacaugcccuaguuagcaagcaagaauaaaaaauuaaaaaaaggggaauuuuuauuuuuuauuuuuuauucuugcuugcuaacuagggcauguagauuugauguuuacaaauucugcuaaucuauuccagaccuuaacuucauaauugauaaaaacaugaauaaauauauuuuagaaaauacuaauuguaaauaggugauaaaaca -3'   exp
    ......((((((((((((((((((((((.((((((((((((.((((((((((((((((((((((((((((((((((.(((((.((((....)))).))))).)))))))))))))))))))))))))))))))))).))))))))))))).))))))))))))).)))))))))))))))))))))))))).......(((....)))......(((..((...((((..((((......)))).)))).))..)))........   reads   mm   sample
    ......................cagaauuucuaaacaucaaaucg.......................................................................................................................................................................................   17   0   LCK-2
    ......................cagaauuucuaaacaucaaaucg.......................................................................................................................................................................................   32   0   LCK-1
    ......................cagaaGuucuaaacaucaaaucg.......................................................................................................................................................................................   1   1   LCK-1
```
